# The Inhibitory Effects and Cytotoxic Activities of the Stem Extract of *Sarracenia purpurea* against Melanoma Cells and the SsbA Protein

**DOI:** 10.3390/plants11223164

**Published:** 2022-11-18

**Authors:** Hong-Wen Liu, Wei-Yu Chiang, Yen-Hua Huang, Cheng-Yang Huang

**Affiliations:** 1 Department of Rheumatology and Immunology, Antai Medical Care Corporation Antai Tian-Sheng Memorial Hospital, Pingtung 928, Taiwan; 2 Department of Biomedical Sciences, Chung Shan Medical University, Taichung City 402, Taiwan; 3 Department of Medical Research, Chung Shan Medical University Hospital, Taichung City 402, Taiwan

**Keywords:** *Sarracenia purpurea*, *Staphylococcus aureus*, SsbA, dihydrokaempferol, oridonin, cytotoxic activity, B16F10 melanoma, SSB, docking

## Abstract

The *Staphylococcus aureus* SsbA protein (SaSsbA) is a single-stranded DNA-binding protein (SSB) that is categorically required for DNA replication and cell survival, and it is thus an attractive target for potential antipathogen chemotherapy. In this study, we prepared the stem extract of *Sarracenia purpurea* obtained from 100% acetone to investigate its inhibitory effect against SaSsbA. In addition, the cytotoxic effects of this extract on the survival, apoptosis, proliferation, and migration of B16F10 melanoma cells were also examined. Initially, myricetin, quercetin, kaempferol, dihydroquercetin, dihydrokaempferol, rutin, catechin, β-amyrin, oridonin, thioflavin T, primuline, and thioflavin S were used as possible inhibitors against SaSsbA. Of these compounds, dihydrokaempferol and oridonin were capable of inhibiting the ssDNA-binding activity of SaSsbA with respective IC_50_ values of 750 ± 62 and 2607 ± 242 μM. Given the poor inhibition abilities of dihydrokaempferol and oridonin, we screened the extracts of *S. purpurea*, *Nepenthes miranda*, and *Plinia cauliflora* for SaSsbA inhibitors. The stem extract of *S. purpurea* exhibited high anti-SaSsbA activity, with an IC_50_ value of 4.0 ± 0.3 μg/mL. The most abundant compounds in the stem extract of *S. purpurea* were identified using gas chromatography–mass spectrometry. The top five most abundant contents in this extract were driman-8,11-diol, deoxysericealactone, stigmast-5-en-3-ol, apocynin, and α-amyrin. Using the MOE-Dock tool, the binding modes of these compounds, as well as dihydrokaempferol and oridonin, to SaSsbA were elucidated, and their binding energies were also calculated. Based on the S scores, the binding capacity of these compounds was in the following order: deoxysericealactone > dihydrokaempferol > apocynin > driman-8,11-diol > stigmast-5-en-3-ol > oridonin > α-amyrin. Incubation of B16F10 cells with the stem extract of *S. purpurea* at a concentration of 100 μg/mL caused deaths at the rate of 76%, reduced migration by 95%, suppressed proliferation and colony formation by 99%, and induced apoptosis, which was observed in 96% of the B16F10 cells. Overall, the collective data in this study indicate the pharmacological potential of the stem extract of *S. purpurea* for further medical applications.

## 1. Introduction

Several ethnobotanical uses for *Sarracenia purpurea* have been noted in many aboriginal communities [1]. For example, the leaf extract of *S. purpurea* is a traditional medicine used for the treatment of type 2 diabetes [2]. This extract also exhibits anti-mycobacterial activity for the treatment of tuberculosis-like symptoms [3]. The root extract of *S. purpurea* has displayed cytotoxic activities against 4T1 mammary carcinoma [4]. It is still unknown whether the stem of *S. purpurea* exhibits cytotoxic activities against cancer cells. In this study, the stem extract of *S. purpurea* was therefore used to test for the suppression of B16F10 melanoma cells and inhibition of the activity of SsbA, an essential DNA binding protein in Gram-positive bacteria [5].

The single-stranded DNA (ssDNA) binding protein (SSB) is essential for all aspects of DNA metabolism, such as DNA replication, repair, recombination, and replication restart [6,7,8,9,10,11]. In these reactions, SSB binds and maintains the transient unwinding of duplex DNA in the single-stranded state [12]. SSBs are well studied in eubacteria, particularly in *Escherichia coli* SSB (EcSSB) [13]. Most, but not all, SSBs are active as homotetramers, in which four oligonucleotide/oligosaccharide-binding folds (OB folds) [14,15] form a DNA-binding domain [16]. In addition to DNA binding, SSB also binds to many DNA-metabolism proteins [17], which constitute the SSB interactome [17,18,19]. Replication protein A (RPA) is the eukaryotic equivalent of bacterial SSB, but RPA and SSB are different in terms of their structure and many other functions [10]. Given that SSB is absolutely required for DNA metabolic processes [20], the pharmacological inhibition of bacterial SSB may be used to target pathogens [21].

Antibiotic-resistant strains of pathogenic bacteria are increasingly prevalent in hospitals and the community [22]. These multidrug-resistant pathogenic bacteria are spreading rapidly worldwide and can become untreatable [23]. As recognized by the Infectious Diseases Society of America, ESKAPE pathogens (*Enterococcus faecium*, *Staphylococcus aureus*, *Klebsiella pneumoniae*, *Acinetobacter baumanii*, *Pseudomonas aeruginosa*, and *Enterobacter* species) cause the majority of US hospital infections and effectively “escape” the effects of antibacterial drugs [24,25]. *S. aureus* exhibits a remarkable ability to develop antibiotic resistance and causes a wide variety of clinical diseases [26]. Methicillin-resistant *S. aureus* (MRSA), one of the three classes of antibiotic-resistant pathogens that are major threats to public health [22], is estimated to cause approximately 19,000 deaths per year in the United States [27]. Thus, the continuous development of clinically useful small-molecule antibiotics is greatly needed in order to target *S. aureus* and treat drug-resistant infections [22,24,28,29,30,31].

EcSSB consists of an N-terminal ssDNA-binding/oligomerization domain (SSBn) and a flexible C-terminal protein–protein interaction domain (SSBc) [18]. EcSSBc can be further subdivided into two sub-domains, i.e., the intrinsically disordered linker and the acidic tip [18]. Different bacterial SSBs share moderate homology, particularly within SSBn (approximately the first 110 residues). SSBn may be expected to be a suitable common target for the design of inhibitors against SSBs [21]. In *S. aureus*, three paralogous SSBs [32], namely, SaSsbA [33], SaSsbB [34,35], and SaSsbC [36], are found. SaSsbA possesses an acidic tip and shares sequence similarity with EcSSBn and is thus referred to as a counterpart of EcSSB [33]. Accordingly, discovering inhibitors against SaSsbA is of considerable interest for further antipathogen applications.

Previously, we found that the flavonol myricetin can bind to and inhibit *P. aeruginosa* SSB (PaSSB) [37,38]. The complexed crystal structure showed that the binding site of the inhibitor myricetin overlapped with the ssDNA-binding sites of PaSSBn [38]. SaSsbA shares structural similarity with EcSSB [33,39] and PaSSB [40,41,42]. Given their structural similarity, one might conclude that the PaSSB inhibitor myricetin must inhibit SaSsbA. In this study, however, we found that myricetin could not inhibit the ssDNA-binding activity of SaSsbA. We then screened for possible SaSsbA inhibitor(s) from plant extracts of *S. purpurea*, *Nepenthes miranda*, and *Plinia cauliflora*. The results from the collective data indicated that the stem extract of *S. purpurea* exhibited significant anti-SaSsbA and anti-B16F10 melanoma activities. The chemical composition of the stem extracts of *S. purpurea* was analyzed via gas chromatography–mass spectrometry (GC–MS). Further studies should directly focus on testing these active ingredients in *S. purpurea* stems for the development of drugs against SaSsbA and B16F10 melanoma and to determine whether and how the stem extract of *S. purpurea* can be used as an alternative treatment.

## 2. Results

### 2.1. Binding of SaSsbA to ssDNA

**Figure 1:**
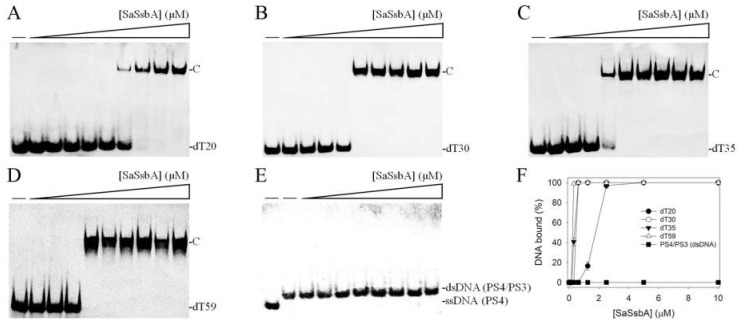
Binding of SaSsbA to ssDNA and dsDNA. Purified SaSsbA (0, 0.039, 0.078, 0.156, 0.312, 0.625, 1.25, 2.5, 5, 10 μM) was incubated with the biotin-labeled ssDNAs (**A**) dT20, (**B**) dT30, (**C**) dT35, and (**D**) dT59 at 37 °C for 60 min. (**E**) Results of binding test of SaSsbA to dsDNA. Purified SaSsbA (0, 0.078, 0.156, 0.312, 0.625, 1.25, 2.5, 5, 10 μM) was incubated with the dsDNA PS4/PS3, of which the DNA strand PS4 was biotinylated. SaSsbA was not able to produce a band shift with this dsDNA. (**F**) ssDNA-binding abilities of SaSsbA. The binding constants ([Protein]_50_) were quantified through linear interpolation based on the protein concentration.

The ssDNA binding ability of SaSsbA was analyzed using an electrophoretic mobility shift assay (EMSA). Different dT homopolymers (dT20, dT30, dT35, and dT59) were biotinylated at the 5′ terminal (Figure 1) and incubated with purified SaSsbA at different concentrations. These biotin-labeled ssDNAs and their complexes could be detected by means of a streptavidin–horseradish peroxidase conjugate. Through EMSA, a significant band shift was observed when SaSsbA was incubated with these ssDNAs. These results indicated that SaSsbA was capable of forming a stable complex with dT20 (Figure 1A), dT30 (Figure 1B), dT35 (Figure 1C), and dT59 (Figure 1D).

To assess whether SaSsbA could bind to double-stranded DNA (dsDNA), the 25 base-pair (bp) dsDNA substrate PS4/PS3 was prepared by annealing two oligonucleotides (PS4 and PS3), of which the DNA strand PS4 was biotinylated. In contrast to dT20 and other ssDNAs, PS4/PS3 incubated with purified SaSsbA at different concentrations was not able to produce a band shift (Figure 1E). Thus, we concluded that SaSsbA could not bind to this dsDNA.

To compare the ssDNA-binding abilities of SaSsbA to these ssDNAs of different lengths, the midpoint values for input ssDNA binding, calculated based on the titration curves of EMSA and referred to as [Protein]_50_ (monomers), were quantified and are summarized in Table 1. According to the titration curves (Figure 1F), the binding constants of SaSsbA to dT20, dT30, dT35, and dT59 were calculated to be 1.77 ± 0.11, 0.46 ± 0.03, 0.36 ± 0.02, and 0.24 ± 0.01 μM, respectively. The formation of the SaSsbA–ssDNA complex was ssDNA-length-dependent, i.e., the longer length of the ssDNA, the higher the binding affinity (Table 1).

### 2.2. Binding of SaSsbA to ssDNA-Containing dsDNA

**Figure 2:**
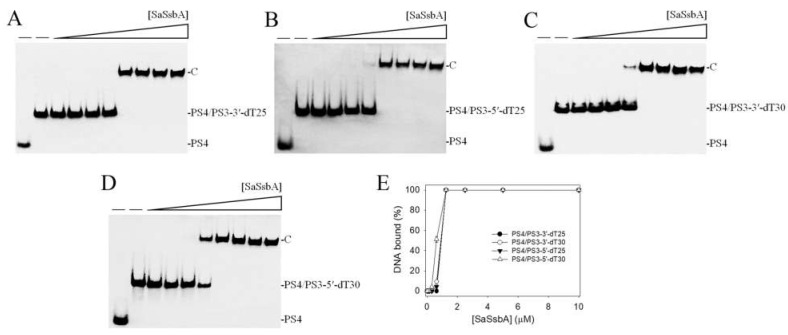
Binding of SaSsbA to dsDNA with ssDNA overhang. Purified SaSsbA (0, 0.078, 0.156, 0.312, 0.625, 1.25, 2.5, 5, 10 μM) was incubated with (**A**) PS4/PS3-3′-dT25, (**B**) PS4/PS3-5′-dT25, (**C**) PS4/PS3-3′-dT30, and (**D**) PS4/PS3-5′-dT30 at 37 °C for 60 min. (**E**) DNA-binding abilities of SaSsbA. The binding constants ([Protein]_50_) were quantified through linear interpolation based on the protein concentration.

No band shift was observed when SaSsbA was incubated with PS4/PS3 (Figure 1E). We further tested whether SaSsbA could bind to dsDNA with ssDNA overhangs of 25 and 30 mer dT. An ssDNA overhang was placed at the 3′ (PS4/PS3-3′-dT25 and PS4/PS3-3′-dT30) or 5′ end (PS4/PS3-5′-dT25 and PS4/PS3-5′-dT30) to investigate the binding preference. Unlike PS4/PS3, the results showed that SaSsbA could produce a band shift with these ssDNA-containing duplex DNAs and form a stable complex. Given that SaSsbA was able to bind to ssDNA dT20–59 (Figure 1), SaSsbA can likely bind to PS4/PS3-3′-dT25 (Figure 2A) and PS4/PS3-5′-dT25 (Figure 2B) because of the dT25 tail in these ssDNA-containing duplex DNAs. Based on the [Protein]_50_ values, the binding of SaSsbA to PS4/PS3-3′-dT25 and PS4/PS3-5′-dT25 was comparable. For comparison, the [Protein]_50_ values of SaSsbA for binding of dT30 (Figure 1B), PS4/PS3-3′-dT30 (Figure 2C), and PS4/PS3-5′-dT30 (Figure 2D) were 0.46 ± 0.03, 0.90 ± 0.04, and 0.62 ± 0.03 μM, respectively (Figure 2E); thus, SaSsbA preferred to bind ssDNA, rather than ssDNA-containing dsDNA. In addition, the binding ability of PS4/PS3-3′-dT30 to SaSsbA was 1.5-fold lower than that of PS4/PS3-5′-dT30, suggesting a certain steric hindrance in the formation of the SaSsbA–PS4/PS3-3′-dT30 complex. Our laboratory is currently attempting to obtain crystals of the SaSsbA–PS4/PS3-3′-dT30 complex for investigation of this phenomenon.

### 2.3. The Flavonol Myricetin, an Inhibitor of PaSSB, Did Not Inhibit SaSsbA

**Figure 3:**
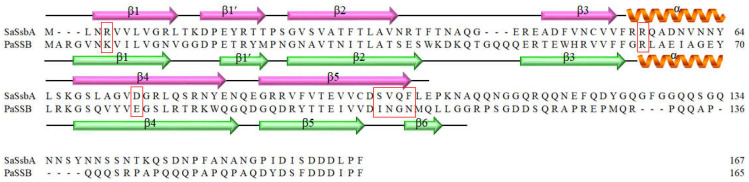
Sequence alignment of SaSsbA and PaSSB. Residues Lys7, Arg62, Glu80, Ile105, Asn106, Gly107, and Asn108 in PaSSB are involved in myricetin binding. The corresponding residues in SaSsbA are indicated by red boxes. Only Arg56 in SaSsbA was conserved as a possible site for myricetin binding. The secondary structural elements of SaSsbA and PaSSB are shown with the sequences.

**Figure 4:**
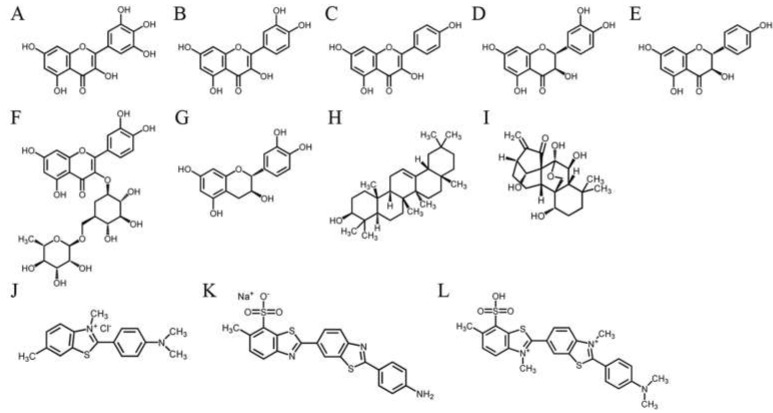
Molecular structure of (**A**) myricetin, (**B**) quercetin, (**C**) kaempferol, (**D**) dihydroquercetin, (**E**) dihydrokaempferol, (**F**) rutin, (**G**) catechin, (**H**) β-amyrin, (**I**) oridonin, (**J**) thioflavin T, (**K**) primuline, and (**L**) thioflavin S. Thioflavin S is a mixture of compounds used as a fluorescent dye to stain Alzheimer’s plaques. The structure of the major component of thioflavin S is shown according to Wu et al. [44].

**Figure 5:**
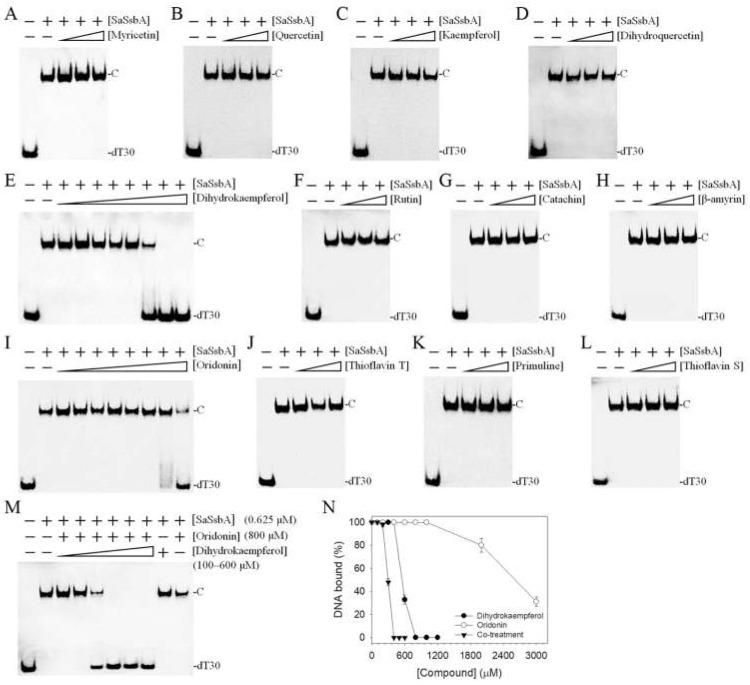
Inhibition of the ssDNA binding activity of SaSsbA. SaSsbA (0.625 μM) was incubated with (**A**) myricetin, (**B**) quercetin, (**C**) kaempferol, (**D**) dihydroquercetin, (**E**) dihydrokaempferol, (**F**) rutin, (**G**) catechin, (**H**) β-amyrin, (**I**) oridonin, (**J**) thioflavin T, (**K**) primuline, and (**L**) thioflavin S. These compounds were dissolved in 10% dimethyl sulfoxide (DMSO). The flavanonol dihydrokaempferol and the diterpenoid oridonin were able to inhibit SaSsbA. (**M**) Co-treatment of oridonin (800 μM) and dihydrokaempferol (0–600 μM) for the inhibition of SaSsbA. (**N**) IC_50_ determinations for SaSsbA.

Given that similar ssDNA-binding domains can be selectively targeted, SSB inhibitors can have various specificities in inhibiting different SSBs [21,43]. Recently, we found that the flavonol myricetin was an inhibitor against PaSSB, with an IC_50_ value of 2.8 μM [37]. Our complexed crystal structure of PaSSB with myricetin further revealed that Lys7, Arg62, Glu80, Ile105, Asn106, Gly107, and Asn108 are involved in myricetin binding (PDB ID 5YUN) [38]. The corresponding residues in SaSsbA are Arg4, Arg56, Asp74, Ser99, Val100, Gln101, and Phe102 (Figure 3), i.e., only Arg56 in SaSsbA is conserved as a possible site for myricetin binding. Structurally, myricetin may not inhibit the ssDNA-binding activity of SaSsbA because the binding residues are significantly different in SaSsbA (Figure 3). An inhibition assay (Figure 4 and Figure 5) was performed to assess whether myricetin is an inhibitor against SaSsbA (Figure 4A and Figure 5A). Other myricetin analogs, the flavonols quercetin (Figure 4B and Figure 5B) and kaempferol (Figure 4C and Figure 5C), bearing different numbers of hydroxyl substituents on the aromatic ring, were also analyzed for their SaSsbA inhibition effects. Each of these flavonols (0–300 μM) was included in the binding assay. Unlike the case in PaSSB, however, even at a concentration of 300 μM, myricetin did not inhibit SaSsbA. Accordingly, we concluded that myricetin, an inhibitor of PaSSB, was not an inhibitor against SaSsbA.

### 2.4. The Flavanonol Dihydrokaempferol and the Diterpenoid Oridonin Were Able to Inhibit SaSsbA

Previously, we found that the flavanonol taxifolin, which is also known as dihydroquercetin, was capable of inhibiting the ssDNA-binding activity of *Salmonella enterica* SSB (SeSSB) [45]. An inhibition assay was also performed to investigate whether dihydroquercetin is an inhibitor of SaSsbA. According to the EMSA, dihydroquercetin did not influence the binding of SaSsbA to ssDNA (Figure 5D), even at 1000 μM (data not shown). Thus, dihydroquercetin is an inhibitor only against SeSSB but not against SaSsbA.

However, dihydrokaempferol, the dihydroquercetin analog, was found to be an inhibitor of SaSsbA (Figure 5E). Based on the titration curve, the IC_50_ value was 750 ± 62 μM. Other flavonoids, rutin (Figure 5F) and catechin (Figure 5G), were also tested but were judged to be noninhibitors. β-amyrin (Figure 5H), a pentacyclic triterpene known as a potential inhibitor of xanthine oxidase [46], tyrosinase [46], and the main protease of SARS-CoV-2 [47,48], could not inhibit SaSsbA. Oridonin (Figure 5I), a natural diterpenoid that is an inhibitor of the Nsp9 protein of SARS-CoV-2 [49], was found to inhibit SaSsbA, with an IC_50_ value of 2607 ± 242 μM. Thioflavin T (Figure 5J), a specific binder of β-amyloid fibrils [50], did not influence the activity of SaSsbA. Primuline (Figure 5K) and thioflavin S (Figure 5L), identified as NS3 helicase inhibitors of the hepatitis C virus [51], were not capable of inhibiting SaSsbA.

We found that the flavanonol dihydrokaempferol and the diterpenoid oridonin could inhibit SaSsbA. Given that the structures of these two natural products are not similar (Figure 4E,I), they might bind to and inhibit SaSsbA in different ways. Accordingly, we investigated whether these two compounds could cooperatively inhibit SaSsbA (Figure 5M). Oridonin at a concentration of 800 μM (a concentration with no inhibition effect on SaSsbA) was selected for this co-treatment experiment. When oridonin was present at a concentration of 800 μM, dihydrokaempferol inhibited SaSsbA with an IC_50_ value of 296 ± 25 μM (Figure 5N). This result might indicate a potential synergistic inhibitory effect, as the co-treatment of dihydrokaempferol with oridonin was able to produce greater inhibition (IC_50_ values from 750 to 296 μM) against SaSsbA (Table 2).

### 2.5. Inhibition of SaSsbA by Plant Extracts

**Figure 6:**
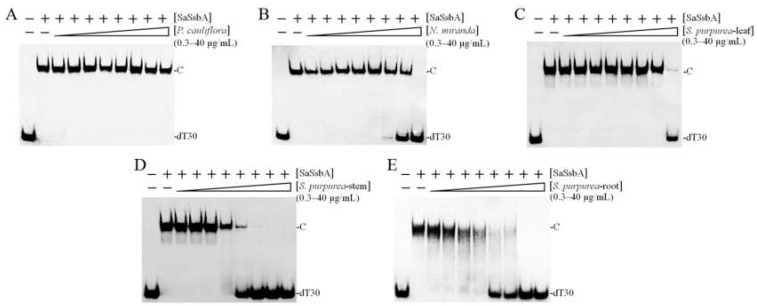
Inhibition of the ssDNA binding activity of SaSsbA by plant extracts. SaSsbA (0.625 μM) was incubated with extracts of (**A**) *P. cauliflora*, (**B**) the stem of *N. miranda*, (**C**) the leaf of *S. purpurea*, (**D**) the stem of *S. purpurea*, and (**E**) the root of *S. purpurea*. These extracts were obtained using 100% acetone. Among these extracts, the stem extract of *S. purpurea* exhibited the greatest inhibitory effect against SaSsbA.

Given the poor inhibition abilities of the compounds used in this study, we screened for new SaSsbA inhibitor(s) from plant extracts. We obtained different acetone extracts from *Plinia cauliflora*, *Nepenthes miranda*, and *Sarracenia purpurea* to determine their possible inhibitory effects against SaSsbA (Figure 6). The *P. cauliflora* extract did not affect SaSsbA activity (Figure 6A). However, the *N. miranda* (Figure 6B) and *S. purpurea* (Figure 6C–E) extracts did inhibit SaSsbA activity (Table 2). The stem extract of *N. miranda* inhibited SaSsbA with an IC_50_ value of 17.6 ± 2.0 μg/mL. The leaf, stem, and root extracts of *S. purpurea* inhibited SaSsbA with IC_50_ values of 34.8, 4.0, and 4.7 μg/mL, respectively. Thus, certain compound(s) in the acetone fraction of the *S. purpurea* extract could be potential SaSsbA inhibitors.

### 2.6. Gas Chromatography–Mass Spectrometry (GC–MS) Analysis of the Stem Extract of S. purpurea

**Figure 7:**
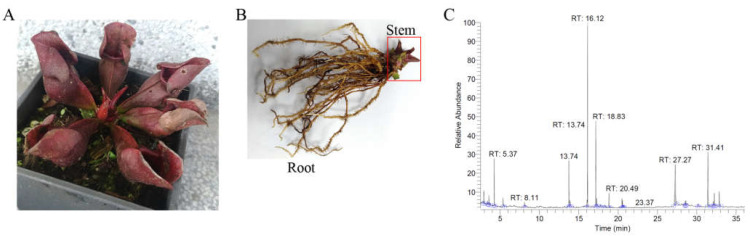
GC–MS analysis of the stem extract of *S. purpurea*. (**A**) *S. purpurea*, a carnivorous pitcher plant with cone-shaped leaves, used for obtaining supplemental nutrients. (**B**) Stem of *S. purpurea*. The stem extract of *S. purpurea* was used for GC–MS analysis. (**C**) GC chromatogram of compounds present in the stem extract of *S. purpurea*. Compounds were identified by matching generated spectra with the NIST 2011 and Wiley 10th Edition mass spectral libraries. The top 5 contents in the stem extract of *S. purpurea* were as follows: driman-8,11-diol, deoxysericealactone, stigmast-5-en-3-ol, apocynin, and α-amyrin.

Given its significant ability to inhibit SaSsbA, the most abundant compounds in the stem extract of *S. purpurea* (Figure 7A,B) were identified using gas chromatography–mass spectrometry (GC–MS). These compounds (Figure 7C) were identified by matching the generated spectra with the NIST 2011 and Wiley 10th Edition mass spectral libraries. The top five contents (>4.7%) in the stem extract of *S. purpurea* were as follows: driman-8,11-diol (18.8%), deoxysericealactone (15.89%), stigmast-5-en-3-ol (12.17%), apocynin (5.94%), and α-amyrin (4.7%). Accordingly, these compounds might be useful alone or in combination as inhibitors of SaSsbA.

### 2.7. Molecular Docking

**Figure 8:**
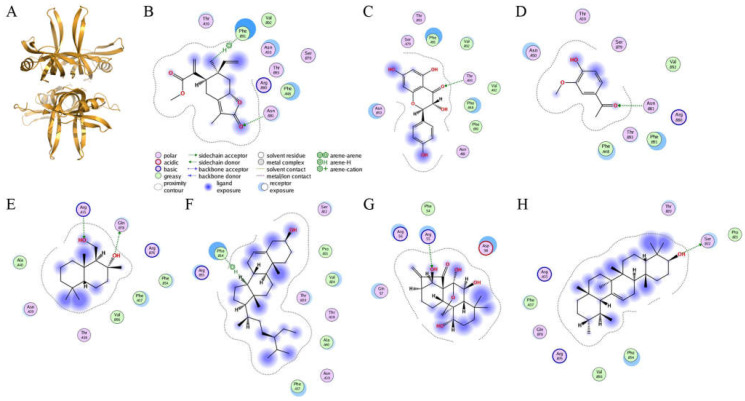
Molecular docking. (**A**) Crystal structure of a tetrameric SaSsbA (PDB ID 5XGT). SaSsbA–ligand binding affinities with all possible binding geometries were predicted on the basis of the S score. Using MOE-Dock software, the binding modes of (**B**) deoxysericealactone, (**C**) dihydrokaempferol, (**D**) apocynin, (**E**) driman-8,11-diol, (**F**) stigmast-5-en-3-ol, (**G**) oridonin, and (**H**) α-amyrin to SaSsbA were predicted.

Given that the stem extract of *S. purpurea* exhibited anti-SaSsbA activity, certain compound(s) in this extract might be responsible for the inhibition of SaSsbA. According to the GC–MS analysis, driman-8,11-diol, deoxysericealactone, stigmast-5-en-3-ol, apocynin, and α-amyrin in the stem extract of *S. purpurea* were identified. Accordingly, we elucidated each compound’s mode of binding to SaSsbA and calculated their binding energies using the Dock tool in Molecular Operating Environment (MOE) software (Figure 8). SaSsbA-ligand binding affinities with all possible binding geometries were predicted on the basis of the docking score (the S score). Dihydrokaempferol and oridonin, inhibitors of SaSsbA (Figure 5), also docked to SaSsbA (PDB ID 5XGT). Based on the S scores (Table 3), the binding capacity of these compounds was in the following order: deoxysericealactone > dihydrokaempferol > apocynin > driman-8,11-diol > stigmast-5-en-3-ol > oridonin > α-amyrin. Deoxysericealactone, possessing the highest S score, exhibited the greatest binding affinity to SaSsbA among these selected compounds.

### 2.8. Cytotoxic Activities against B16F10 Melanoma Cells

**Figure 9:**
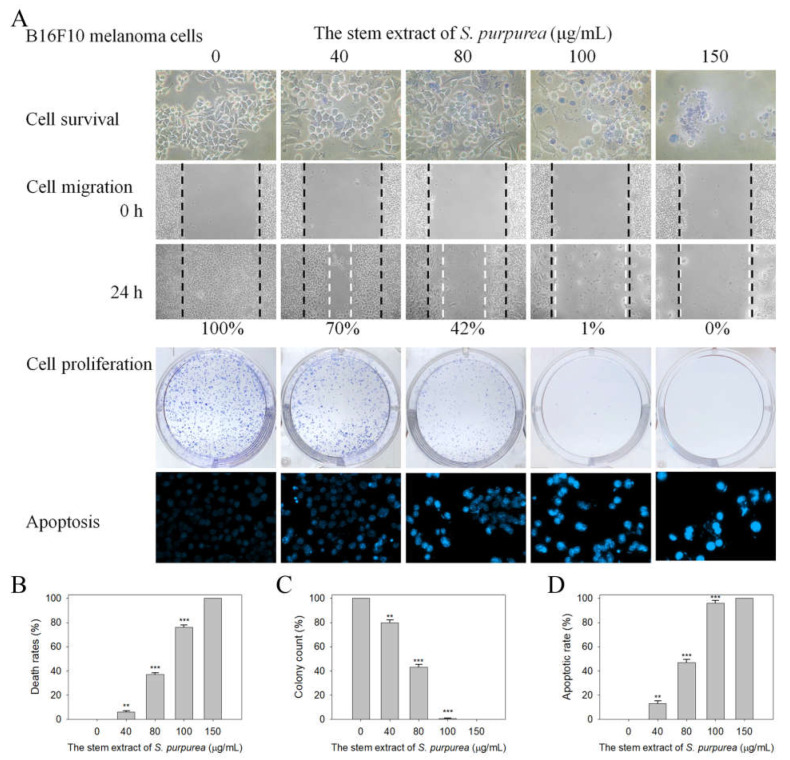
The cytotoxic effects of the stem extract. (**A**) Effects on cell survival, migration and proliferation, and apoptosis. (**B**) Trypan blue dye exclusion staining results. B16F10 cells incubated with the stem extract of *S. purpurea* at concentrations of 40, 80, 100, and 150 μg/mL. (**C**) Clonogenic formation assay results. Pretreatment with the stem extract of *S. purpurea* significantly suppressed the proliferation and colony formation of B16F10 cells. (**D**) Hoechst staining results. Apoptosis induced by the stem extract of *S. purpurea* with DNA fragmentation was observed in B16F10 cells. ** *p* < 0.01 and *** *p* < 0.001 compared with the control group.

The question of whether the stem extract of *S. purpurea* exhibited cytotoxic activities against B16F10 melanoma cells was also investigated (Figure 9). In addition to the SaSsbA inhibition capacity, we found that the stem extract of *S. purpurea* also exhibited cytotoxicity on melanoma cell survival, migration, and proliferation, and also induced cell apoptosis (Figure 9A). The death rate of B16F10 cells caused by the stem extract of *S. purpurea* was estimated using a trypan blue staining assay after 0 and 24 h of incubation (Figure 9B). Incubation with the stem extract of *S. purpurea* at concentrations of 40, 80, 100, and 150 μg/mL caused the deaths of B16F10 cells at the rates of 6%, 37%, 76%, and 100%, respectively. According to the wound-healing assay, the stem extract of *S. purpurea* strongly reduced the migration of B16F10 cells. After 24 h of incubation, the stem extract of *S. purpurea* at concentrations of 40, 80, 100, and 150 μg/mL inhibited B16F10 cell migration by 30%, 58%, 95%, and 100%, respectively. The cytotoxic effects of the stem extract of *S. purpurea* on the proliferation (Figure 9C) and apoptosis (Figure 9D) of B16F10 cells were also examined. A clonogenic formation assay revealed that pretreatment with the stem extract at a concentration of 100 μg/mL significantly suppressed the proliferation and colony formation of B16F10 cells (99%). Hoechst staining showed stem extract (100 μg/mL)-induced apoptosis with DNA fragmentation in 96% of the B16F10 cells. Thus, the stem extract of *S. purpurea* exhibited cytotoxic activities against B16F10 melanoma cells.

### 2.9. The Stem Extract Suppressed Melanoma Cell Proliferation by Inducing G2 Cell-Cycle Arrest

**Figure 10:**
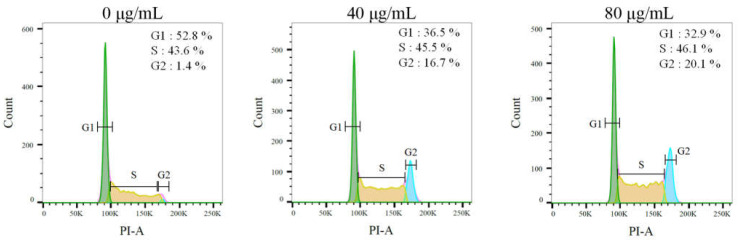
Flow analysis results. B16F10 cells were treated with the stem extract of *S. purpurea* at indicated concentrations for 24 h and fixed with 70% alcohol overnight. The cell suspension was stained with propidium iodide (PI) for 30 min and subjected to flow cytometry.

We examined the effect of the stem extract against the cell-cycle progression of melanoma cells by means of flow cytometry (Figure 10). The B16F10 cells were treated with the stem extract of *S. purpurea* at concentrations of 40 and 80 μg/mL. The stem extract increased the count of double DNA content cells in a concentration-dependent manner. The stem extract of *S. purpurea* boosted the distribution of the G2 phase from 1.4% to 16.7% at a concentration of 40 μg/mL and to 20.1% at a concentration of 80 μg/mL in the B16F10 cells. Thus, the stem extract might suppress melanoma cell proliferation by inducing G2 cell-cycle arrest.

## 3. Discussion

The purple carnivorous pitcher plant *S. purpurea* [4] is a medicinal plant, used by Canadian First Nations people to treat a wide variety of illnesses [1]. Due to its longstanding ethnomedicinal uses, the extracts of *S. purpurea* are safe as pharmaceuticals and are expected to have few side effects for human use. In this study, we found that the stem extract of *S. purpurea* exhibited anti-SaSsbA activity (Figure 5 and Figure 6) and anticancer potential (Figure 9 and Figure 10). Suppression of DNA replication and metabolism is widely used as an antimicrobial strategy for antibiotic design. For example, quinolone and aminocoumarin antibiotics were successfully developed to target DNA gyrase and topoisomerase IV [52,53] for antipathogen chemotherapy. Given that SSB is absolutely required for DNA replication [20], the pharmacological inhibition of bacterial SSB may be used to target pathogens [21]. Like SSB, many nucleic acid-binding proteins possess OB-fold domain(s) [54]. OB-fold-containing proteins are currently recognized as druggable targets for oncology and drug discovery [54]. For example, the OB-fold domain in the breast cancer susceptibility protein BRCA2 represents an attractive cancer drug target [55]. The modes of inhibition of SaSsbA by these small molecules (Figure 8) in regard to the drugging of these binding sites in OB-fold domain(s) may also provide insights into how these inhibitors, such as myricetin [56,57] and quercetin [58,59,60,61], which are known as potential cancer therapeutics, can bind and inhibit other OB-fold proteins in cancer-signaling pathways [37,38,45,62]. Thus, it is of considerable interest to continue to search for inhibitors against OB-fold-containing proteins.

Similarly to the carnivorous pitcher plant *N. miranda* [63,64], *S. purpurea* also exhibited cytotoxicity in regard to cancer cell survival, migration, and proliferation. However, their ingredients, as identified via GC-MS, were found to be significantly different [64,65]. The preliminary data in this study indicated that the stem extract of *S. purpurea* could be a potential natural alternative or complementary therapy for melanoma cancer. The top five components (>4.7%) found in the stem extract of *S. purpurea* (Figure 7) were as follows: driman-8,11-diol (18.8%), deoxysericealactone (15.89%), stigmast-5-en-3-ol (12.17%), apocynin (5.94%), and α-amyrin (4.7%). These compounds might be useful alone or in combination by exerting cytotoxic effects on melanoma cells and as inhibitor(s) of SaSsbA.

Many SSB proteins bind to ssDNA with some degree of positive cooperativity [13]. According to the EMSA, SSB proteins form multiple distinct complexes with ssDNAs of different lengths, such as PaSSB [40,41,42], SeSSB [66], *K. pneumonia* SSB (KpSSB) [67,68], DnaD [69,70], and DnaT [71,72]. The EMSA, a popular and well-established approach in studies of molecular biology, can detect distinct protein–DNA complexes [73]. These proteins bind ssDNAs with lengths > 55 nt and form a second distinct complex. In contrast, SaSsbA binding to ssDNAs of different lengths only forms a single complex (Table 1 and Figure 1). The distinct second complex was not observed even when dT59 was used. This EMSA behavior of SaSsbA resembles that of PriB [74,75] and the DnaT84-179 protein [76]. Similarly to SaSsbA (Figure 1), PriB binds ssDNAs of different lengths and only forms a single complex. The ssDNA binding patterns of SaSsbA did not resemble those of PaSSB, SeSSB, and KpSSB; thus, SaSsbA may bind ssDNA in a manner that is different from that of Gram-negative bacterial SSBs. Interestingly, *ssbA* (*S. aureus*) and *priB* (*K. pneumonia* and *E. coli*) are coincidentally embedded within the same ribosomal protein operon (*rpsF* and *rpsR*) [77] and controlled by the SOS response [5]. That is, the respective main *ssb* genes in the Gram-positive and -negative bacteria are located far apart and embedded within different operons. This fact may, therefore, provide a clue regarding the binding similarity to that of ssDNA. However, the degree of similarity of the ssDNA binding mode of SaSsbA to PriB should be further demonstrated experimentally and structurally.

*S. aureus* is a Gram-positive pathogen that exhibits a remarkable ability to develop antibiotic resistance [22,26]. DNA metabolism, such as the processes mediated by SSB, is one of the most basic biological functions and should be a prime target in antibiotic development [21]. In this study, we found that the flavanonol dihydrokaempferol and the diterpenoid oridonin were able to inhibit the ssDNA binding activity of SaSsbA (Figure 5 and Table 2). Dihydrokaempferol is also a competitive inhibitor of monophenolase and diphenolase [78,79]. Oridonin is an inhibitor against both the main protease and the Nsp9 protein of SARS-CoV-2 [49,80]. The combination of oridonin and TRAIL was also found to induce apoptosis in uveal melanoma cells [81]. Nsp9 also possesses an OB-fold domain [82] and was therefore selected as a test compound for the inhibition of SaSsbA (Figure 5). To understand its binding site(s), our laboratory attempted to obtain crystals of SaSsbA and Nsp9 in a complex with oridonin for crystallographic analysis so as to compare their inhibition modes.

Flavonoids have several hydroxyl groups and thus have significant antioxidant activity and a marked potential for binding proteins. Myricetin, an inhibitor of PaSSB [37,38], could also have been expected to be an inhibitor against SaSsbA. However, myricetin was not capable of inhibiting SaSsbA (Figure 5). Dihydroquercetin, an inhibitor of SeSSB, also did not influence the binding of SaSsbA to ssDNA. Thus, the bacterial DNA-binding domain of SSBs can be selectively targeted, as previously reported in mammalian systems [43]. To achieve these inhibition modes, the crystal structure of SaSsbA in a complex with these compounds is highly desired.

Unlike Gram-negative bacteria (e.g., *E. coli*), which contain only one type of SSB, Gram-positive bacteria have more than one paralogous SSB [5,32], such as SsbA [33], SsbB [34,35], and SsbC [36] in *S. aureus*. Their structures for binding ssDNA are similar. Although the N-terminal ssDNA-binding domains of *S. aureus* SSBs are structurally similar, a minor sequence difference would result in different inhibitor binding specificities, as was observed with myricetin (Figure 3 and Figure 5). Thus, SaSsbA, SaSsbB, and SaSsbC may have different inhibition specificities and can be also selectively targeted.

Natural products have been a source of medicinal products for millennia [83]. Natural products or their derivatives account for over one third of the small-molecular drugs approved by the Food and Drug Administration (FDA) [84]. Considering that many natural products exhibit anticancer properties towards skin cancers [85,86], we investigated and found that the stem extract of *S. purpurea* was capable of inhibiting the growth, invasion, and proliferation of B16F10 melanoma cells (Figure 9). Cancer progression is associated with the dysfunction of checkpoint controls, which regulate normal passage through the cell cycle [87]. The G2 cell-cycle checkpoint [88] is a critical genome guardian of tumor cells, and therefore G2 checkpoint abrogation has been considered to be a promising therapeutic anticancer target [87]. Treatment using the stem extract of *S. purpurea* (Figure 10) was found to be able to promote the distribution of the G2 phase and decreased the cell proportion in the G1 and S phases in a concentration-dependent manner in B16F10 melanoma cells. The stem extract might therefore suppress melanoma cell proliferation by inducing G2 cell-cycle arrest. The cellular signaling pathways that trigger this G2 arrest in B16F10 melanoma cells should be investigated further.

In conclusion, this study was the first to identify the anti-SaSsbA effects and cytotoxic activities exerted by the stem extract of *S. purpurea* on the survival, apoptosis, and migration of melanoma cells. The abundant ingredients in this extract were determined via GC–MS to obtain a better understanding of which compound(s) may be active, alone or in combination, in these biological activities. Myricetin and dihydroquercetin, inhibitors of other SSBs, were found not to be inhibitors against SaSsbA. Thus, the bacterial DNA-binding domains of SSBs can be selectively inhibited and may be suitable targets for drug development. These results may indicate the potential of the stem extract of *S. purpurea* for further medical applications.

## 4. Materials and Methods

### 4.1. Chemicals, Cell Line, and Bacterial Strains

All chemicals were purchased from Sigma-Aldrich (St. Louis, MO, USA) and were of analytical grade. All restriction enzymes and DNA-modifying enzymes were purchased from New England Biolabs (Ipswich, MA, USA). The *Escherichia coli* strains TOP10F’ (Invitrogen, CA, USA) and BL21(DE3) pLysS (Novagen, MA, UK) were used for genetic construction and protein expression, respectively. The B16F10 murine melanoma cell lines were obtained from the Food Industry Research and Development Institute, Hsinchu, Taiwan [63,64]. B16F10 cells were cultured in Dulbecco’s modified Eagle’s medium (DMEM) and incubated at 37 °C in a humidified incubator with 5% CO_2_. Medium was supplemented with 10% fetal bovine serum (FBS), 100 unit/mL penicillin, and 100 μg/mL streptomycin.

### 4.2. Recombinant Protein Expression and Purification

The construction of the SaSsbA expression plasmid has been reported previously [33]. The expression vector pET21b–SaSsbA was transformed into *E. coli* BL21 (DE3) cells and grown in LB medium at 37 °C. Overexpression was induced by incubating it with 1 mM isopropyl thiogalactopyranoside for 9 h at 25 °C. The SaSsbA protein was purified from the soluble supernatant via Ni^2+^-affinity chromatography (HisTrap HP; GE Healthcare Bio-Sciences, Piscataway, NJ, USA), eluted with Buffer A (20 mM Tris-HCl, 200 mM imidazole, and 0.5 M NaCl, pH 7.9), and dialyzed against a dialysis buffer (20 mM HEPES and 100 mM NaCl, pH 7.0; Buffer B). Protein purity remained at >97%, as determined via SDS-PAGE (Mini-PROTEAN Tetra System; Bio-Rad, Hercules, CA, USA).

### 4.3. Preparation of dsDNA Substrates

The dsDNA substrate was prepared with a biotinylated PS4 strand (3′-GGGCTTAAGCCTATCGAGCCATGGG-5′; 25 mer) and an unlabeled PS3 strand (5′-CCCGAATTCGGATAGCTCGGTACCC-3′; 25 mer) at a 1:1 concentration ratio (PS4/PS3). Other dsDNA substrates were also prepared with a biotinylated PS4 and an unlabeled PS3-3′-dT25 strand (5′-CCCGAATTCGGATAGCTCGGTACCC-dT25-3′), PS3-5′-dT25, PS3-3′-dT30, or PS3-5′-dT30 for binding comparisons. Each dsDNA substrate was formed in 20 mM HEPES (pH 7.0) and 100 mM NaCl, via brief heating at 95 °C for 5 min, followed by slow cooling to room temperature overnight.

### 4.4. EMSA

Different dT homopolymers (dT20, dT30, dT35, and dT59) were biotinylated at the 5′ terminal and incubated with purified SaSsbA of different concentrations (0–10 μM; 0, 0.039, 0.078, 0.156, 0.312, 0.625, 1.25, 2.5, 5, and 10 μM). Different dsDNA substrates (PS4/PS3, PS4/PS3-3′-dT25, PS4/PS3-5′-dT25, PS4/PS3-3′-dT30, and PS4/PS3-5′-dT30) were also used for EMSA. The final concentration of these DNA substrates for analysis was 30 fmol/μL. EMSA was performed in accordance with a previously described protocol for SeSSB [45] and PaSSB [37] using a LightShift Chemiluminescent EMSA Kit (Thermo Scientific, MA, USA). In brief, SaSsbA was incubated for 60 min at 37 °C with the DNA substrate at a total volume of 6 μL in 40 mM Tris–HCl (pH 7.5) and 50 mM NaCl. Following incubation, 4 μL of a dye mixture (0.01% bromophenol blue and 40% glycerol) was added. Native polyacrylamide gel (8%) was pre-electrophoresed at 110 V for 10 min. Thereafter, the resulting samples were loaded and resolved on pre-run gel and electrophoresed at 100 V for 1 h in TBE running buffer (89 mM Tris borate and 1 mM EDTA). The protein–DNA complexes were electroblotted to positively charged nylon membrane (GE, USA) at 100 V for 30 min in fresh and cold TBE buffer. Transferred DNA was cross-linked with a nylon membrane using a UV-light cross-linker instrument equipped with 312 nm bulbs for a 10 min exposure. Biotin-labeled DNA was detected using streptavidin–horseradish peroxidase conjugate and chemiluminescent substrate contained in SuperSignal™ West Atto Ultimate Sensitivity Substrate (Pierce Biotechnology, Waltham, MA, USA). The DNA-binding ability of SaSsbA was estimated through linear interpolation based on the concentration of the protein that bound 50% of the input DNA.

### 4.5. Inhibition Assay

The EMSA, for the testing of inhibition against SaSsbA, was conducted in accordance with a previously described protocol for SeSSB [45]. Biotinylated dT30 was used as substrate for this inhibition assay. SaSsbA (0.625 μM) was incubated with the indicated compound (0–300 μM) and dT30 for 60 min at 37 °C. Following incubation, the resultant SaSsbA solution was analyzed via the EMSA using a LightShift Chemiluminescent EMSA Kit. Dose-response curves were generated by titrating the compound into the assay solution. The concentration of the compound required for 50% inhibition (IC_50_) was determined directly based on graphical analysis [89,90].

### 4.6. Plant Materials and Extract Preparations

Stems of *S. purpurea* were collected, dried, cut into small pieces, and pulverized into powder. Extractions were carried out by placing 1 g of plant powder into 250 mL conical flask. The flask was added with 100 mL of acetone and shaken on an orbital shaker for 5 h. The resultant extract was filtered using a 0.45 μm filter and stored at −80 °C until use.

### 4.7. GC-MS Analysis

GC-MS analysis was performed to determine the molecular composition of samples. The filtered sample was analyzed using a Thermo Scientific TRACE 1300 Gas Chromatograph with a Thermo Scientific ISQ Single Quadrupole Mass Spectrometer system. The column used was Rxi-5ms (30 m × 0.25 mm i.d. × 0.25 μm film). Helium was used as the carrier gas at a constant flow rate of 1 mL/min. The initial oven temperature was 40 °C and it was maintained at this temperature for 3 min; the temperature was gradually increased to 300 °C at a rate of 10 °C/min and this was maintained for 1 min. The temperature of the injection port was 300 °C and the flow rate of helium was 1 mL/min. The compounds discharged from the column were detected using a quadrupole mass detector. The ions were generated using the electron ionization method. The temperatures of the MS quadrupole and source were 150 °C and 300 °C, respectively; the electron energy was 70 eV; the temperature of the detector was 300 °C; the emission current multiplier voltage was 1624 V; the interface temperature was 300 °C; and the mass range was from 29 to 650 amu. The relative mass fraction of each chemical component was determined via the peak area normalization method. Compounds were identified by matching the generated spectra with the NIST 2011 and Wiley 10th Edition mass spectral libraries.

### 4.8. Trypan Blue Cytotoxicity Assay

The trypan blue cytotoxicity assay was performed to assess cell death [91]. B16F10 cells (1 × 10^4^) were incubated with the extract of *S. purpurea* at a volume of 100 μL [63,64]. After 24 h, the cytotoxic activity exhibited by the extract was estimated by performing trypan blue staining analysis.

### 4.9. Chromatin Condensation Assay

Apoptosis in B16F10 cells was analyzed via Hoechst 33342 staining [92]. B16F10 cells were seeded in 6-well plates at a density of 5 × 10^3^ cells per well in a volume of 200 μL of culture medium. Cells were allowed to adhere for 16 h. After treatment with the extract of *S. purpurea*, cells were incubated for an additional 24 h, washed with PBS and stained with the Hoechst dye (1 μg/mL) in the dark at RT for 10 min. Cells were imaged using the ImageXpress Pico system (Molecular Devices, CA, USA). Image acquisition was performed on each well using 20× magnification and a 6 × 6 square image scan with DAPI filter cubes [65]. Image analyses were performed on the images obtained from the ImageXpress Pico instrument (Molecular Devices, CA, USA) using CellReporterXpress Version 2 software. The apoptotic index was calculated as follows: apoptotic index = apoptotic cell number/(apoptotic cell number + nonapoptotic cell number).

### 4.10. Clonogenic Formation Assay

A clonogenic formation assay [63,93] was used to assess the inhibition of B16F10 cell proliferation. Briefly, B16F10 cells were seeded at a density of 1 × 10^3^ cells per well into 6-well plates and incubated overnight for attachment. The resultant plates were incubated with the extract of *S. purpurea* for 5–7 days to allow clonogenic growth. After washing with PBS, colonies were fixed with methanol and stained with 0.5% crystal violet for 20 min, and the number of colonies was counted under a light microscope.

### 4.11. Wound-Healing Assay

An in vitro migration (wound-healing) assay [63,94] was performed to analyze the inhibition of B16F10 cell migration. Briefly, B16F10 cells were seeded in 24-well plates, incubated in serum-reduced medium for 6 h, wounded in a line across the well with a 200 μL pipette tip, and washed twice with the serum-reduced medium. After treating them with the extract of *S. purpurea*, cells were incubated for 24 h to allow migration.

### 4.12. MOE-Dock Analysis

Through MOE-Dock [95], deoxysericealactone, dihydrokaempferol, apocynin, driman-8,11-diol, stigmast-5-en-3-ol, oridonin, and α-amyrin were analyzed and docked to assess their binding capacity in SaSsbA. Before starting the docking process, the water molecules present in the crystal structure of SaSsbA (PDB ID 5XGT) [33] were removed via MOE. Hydrogen atoms were added to the protein structure through 3D protonation with subsequent minimization of energy. Top-ranked conformations were developed and analyzed.

### 4.13. Flow Analysis

Cell cycle analysis was performed via flow cytometry. B16F10 cells were treated with DMSO or the extract of *S. purpurea* for 24 h and harvested with trypsin. Harvested cells were washed, resuspended in PBS with 1% FBS, and fixed with cold ethanol (70%). Fixed cells were washed, incubated in PBS buffer for 5 min, and resuspended in PI/RNase solution (PBS, RNase, and 50 μg/mL PI) for staining. The resultant cells were stained for 30 min at 37 °C in the dark and analyzed via flow cytometry with a BD FACSCanto II system (BD Biosciences, San Jose, CA, USA). The distribution of each phase was calculated and visualized directly via FlowJo v10 software (Tree Star, Inc., Ashland, OR, USA).

## Figures and Tables

**Table 1:** ssDNA binding properties of SaSsbA as analyzed via EMSA.

DNA	[Protein]_50_ (μM)	Complex Number
dT20	1.77 ± 0.11	1
dT30	0.46 ± 0.03	1
dT35	0.36 ± 0.02	1
dT59	0.24 ± 0.01	1
PS4/PS3	ND	0
PS4/PS3-3′-dT25	0.94 ± 0.05	1
PS4/PS3-3′-dT30	0.90 ± 0.04	1
PS4/PS3-5′-dT25	0.92 ± 0.04	1
PS4/PS3-5′-dT30	0.62 ± 0.03	1

**Table 2:** Inhibition of the ssDNA binding activity of SaSsbA.

Inhibitor	IC_50_
Dihydrokaempferol	750 ± 62 μM
Oridonin	2607 ± 242 μM
Dihydrokaempferol with oridonin (800 μM)	296 ± 25 μM
Extract of *Plinia cauliflora*	N.D.
Stem extract of *Nepenthes miranda*	17.6 ± 2.0 μg/mL
Leaf extract of *Sarracenia purpurea*	34.8 ± 2.6 μg/mL
Stem extract of *Sarracenia purpurea*	4.0 ± 0.3 μg/mL
Root extract of *Sarracenia purpurea*	4.7 ± 0.3 μg/mL

**Table 3:** Results of the docking studies against SaSsbA.

	S Score	Receptor Residue	Interaction	Distance (Å)	E (kcal/mol)
Deoxysericealactone	−5.0401	Asn 81 (B)	H-acceptor	3.29	−0.5
		Phe 91 (B)	H-Pi	3.76	−0.8
Dihydrokaempferol	−4.9087	Thr 93 (A)	H-acceptor	2.73	−1.8
Apocynin	−4.5017	Asn 81 (B)	H-acceptor	3.15	−0.8
Driman-8,11-diol	−4.3941	Gln 78 (B)	H-donor	2.99	−1.6
		Arg 35 (B)	H-acceptor	3.00	−3.3
Stigmast-5-en-3-ol	−4.3728	Phe 54 (B)	H-Pi	4.30	−0.5
Oridonin	−4.1062	Asn 50 (A)	H-acceptor	3.40	−0.8
α-Amyrin	−3.2853	Ser 22 (B)	H-donor	2.85	−1.0

## Data Availability

Not applicable.
